# Probiotic *Pediococcus pentosaceus* ameliorates MPTP-induced oxidative stress *via* regulating the gut microbiota–gut–brain axis

**DOI:** 10.3389/fcimb.2022.1022879

**Published:** 2022-11-09

**Authors:** Sipei Pan, Hongming Wei, Shushu Yuan, Yu Kong, Huiqun Yang, Yuhe Zhang, Xiaorui Cui, Weian Chen, Jiaming Liu, Yang Zhang

**Affiliations:** ^1^ Department of Neurology, The First Affiliated Hospital of Wenzhou Medical University, Wenzhou, Zhejiang, China; ^2^ Department of Geriatrics, The Second Affiliated Hospital and Yuying Children’s Hospital of Wenzhou Medical University, Wenzhou, Zhejiang, China; ^3^ Department of Preventive Medicine, School of Public Health and Management, Wenzhou Medical University, Wenzhou, Zhejiang, China

**Keywords:** Parkinson’s disease, gut microbiota, *P. pentosaceus*, oxidative stress, Nrf2 signaling, γ-aminobutyric acid

## Abstract

Recent evidence demonstrated that functional bacteria were involved in the regulation of Parkinson’s disease (PD). However, the mechanism of probiotics in improving PD was unclear. Here the antioxidant effect and the mechanism of probiotics *Pediococcus pentosaceus* (PP) on PD were studied by regulating the gut–brain axis. In this study, male C57BL/6J mice were injected with 1-methyl-4-phenyl-1,2,3,6-tetrahydropyridine (MPTP) intraperitoneally to establish a PD model and were then treated with PP for 4 weeks. Subsequently, a series of neurobehavioral tests to evaluate the motor function of the mice was performed. Additionally, degeneration of dopaminergic neurons, accumulation of α-synuclein, the production of an oxidative stress response, and the expression of nuclear factor erythroid 2-related factor 2 (Nrf2) pathway-related proteins were evaluated. Moreover, the gut microbial composition and the level of metabolite γ-aminobutyric acid (GABA) were assessed. The results showed that PP treatment could improve MPTP-induced motor deficits, the degeneration of dopaminergic neurons, and the accumulation of α-synuclein. Moreover, PP treatment significantly increased the levels of SOD1, Gpx1, and Nrf2, while it decreased the levels of Keap1 in the brain of MPTP-induced mice. Notably, PP treatment improved the gut microbial dysbiosis and increased the level of GABA in MPTP-induced mice. These findings indicated that PP might represent a promising candidate, due to the metabolite of GABA, that could be used for the treatment of PD.

## Introduction

Parkinson’s disease (PD), the second largest neurodegenerative disease with an increasing prevalence (Dorsey et al., 2018; Marino et al., 2020), is dominated by motor symptoms attributed to the aggregation of α-synuclein and the injury of dopaminergic neurons in the substantia nigra. Current disease-modifying therapies, basically dopamine replacement, have been used in the treatment of PD, but these therapies lack the ability to restrain the degeneration and death of dopaminergic neurons (Raza et al., 2019; Armstrong and Okun, 2020). The neurodegeneration of PD involved many mechanisms, among which oxidative stress played an undeniable role in the loss of dopaminergic neurons by inducing unbalanced redox homeostasis, ultimately leading to neuronal oxidative stress (Puspita et al., 2017; Trist et al., 2019). The nuclear factor erythroid-2 related factor 2 (Nrf2), a key transcription factor in cellular response to oxidative stress, could maintain intracellular redox homeostasis by stimulating the expression of antioxidant and cytoprotective genes (Rojo de la Vega et al., 2018). The Nrf2 pathway can effectively scavenge reactive oxygen species (ROS) and free radicals and protect the dopaminergic neurons from oxidative damage (Wei et al., 2020). Nrf2 exerted its redox-regulating capacities by uncoupling with endogenous inhibitor Keap1 and releasing Nrf2, which was migrated to the nucleus and bound to the promoter region of antioxidant response element (ARE) (Zgorzynska et al., 2021) so as to regulate numerous cytoprotective genes, thus mitigating the oxidative damage and dysfunction of dopaminergic neurons (Ishii et al., 2019). Therefore, regulating the Nrf2 signaling pathway is the key to reduce the oxidative stress of PD.

γ-Aminobutyric acid (GABA), a kind of inhibitory neurotransmitter, could maintain intracellular redox homeostasis and protect neurons from oxidative damage (Ngo and Vo, 2019). Mounting evidence showed that the concentration of GABA was decreased in many neuropsychiatric diseases (Zheng et al., 2017; Cortès-Saladelafont et al., 2018), and there was a negative correlation between the GABA level in the cerebral cortex and the severity of PD symptoms (van Nuland et al., 2020). It was reported that GABA treatment can increase the expression of Nrf2 protein in the nucleus of myoblasts and regulate the level of glutathione and glycogen synthase kinase-3β phosphorylates as well as the activity of catalase (CAT), superoxide dismutase (SOD), and ROS scavenging (Choe et al., 2021) and prevent the H_2_O_2_-induced transfer of Nrf2 into the nucleus, thus further alleviating oxidative stress injury and restoring the redox homeostasis of cells (Tang et al., 2018). Moreover, GABA can reverse the H_2_O_2_-induced mRNA expression and protein expression of Keap1 and Nrf2 and protect endothelial cells from oxidative stress damage by regulating the Nrf2 signaling pathway (Zhu et al., 2019). PD patients were often observed to have gut microbiota dysbiosis (Hill-Burns et al., 2017; Aho et al., 2019), accompanied by degeneration of dopaminergic neurons and decrease of dopamine level in the brain (Koutzoumis et al., 2020). Gut functional bacteria can interact with the host through the production of functional metabolites, which contributes to an in-depth understanding of the impact of gut functional bacteria on the host. Interestingly, many intestinal commensal bacteria, such as *Lactobacillus* and *Bifidobacterium*, can produce GABA (Dinan et al., 2013). This randomized, double-blind trial found that treatment with probiotics (GABA-producing bacteria) for 4 weeks significantly improved the gastrointestinal symptoms in patients with PD. Remarkably, many special probiotics have been proven to inhibit brain dysfunction and motor disorder and increase the expression of antioxidant enzymes in brain tissue, which may prevent the pathological development of PD. Bravo et al. reported that *Lactobacillus rhamnosus* can act on GABA nervous system (Bravo et al., 2011) and improve stress- and anxiety-like behaviors along with the regulation of GABA receptor expression in mice brain (Bravo et al., 2011). *Pediococcus pentosaceus* (PP), belonging to GAB- producing bacteria, was identified as a potential probiotic (Jiang et al., 2021) and had many beneficial effects, including anti-oxidation, anti-inflammatory effect, and ability in reversing abnormal gut microbiota (Jiang et al., 2021; Yu et al., 2021; Dong et al., 2022). So far, the effect of PP against the oxidative stress of PD is still unclear.

In the present study, we explored the beneficial effects of PP against 1-methyl-4-phenyl-1,2,3,6-tetrahydropyridine (MPTP)-induced motor dysfunction *via* regulating the gut–brain axis. Behavioral tests, such as pole test, rotational test, and beam walking test, were evaluated. In addition, the degeneration of dopaminergic neurons was evaluated by measuring the expression of TH and the accumulation of α-synuclein. The activity of antioxidant enzymes and the expression levels of the Nrf2 signaling pathway and downstream-related proteins in brain tissue were detected to evaluate the changes of oxidative stress in the brain of PD mice. Meanwhile, the level of GABA in brain tissue was detected, and the composition and the structural changes of the gut microbiota were evaluated by 16s rRNA sequencing. Our results reveal that GABA-producing bacteria PP might be a novel dietary supplementation of probiotic for inhibiting oxidative stress associated with PD.

## Materials and methods

### Animals

Male C57BL/6J mice (6–8 weeks old and 20–25g in weight) were purchased from Hangzhou Ziyuan Experimental Technology Co, Ltd, Hangzhou, China. The mice were housed in constant temperature (23 ± 2°C) and humidity (55 ± 5%) in a room with 12-h light/dark cycle. Food and water were available *ad libitum*. All animal procedures were performed in accordance with the guidelines of the Animal Ethics Committee of Wenzhou Medical University.

### Experimental design

**Figure 1:**
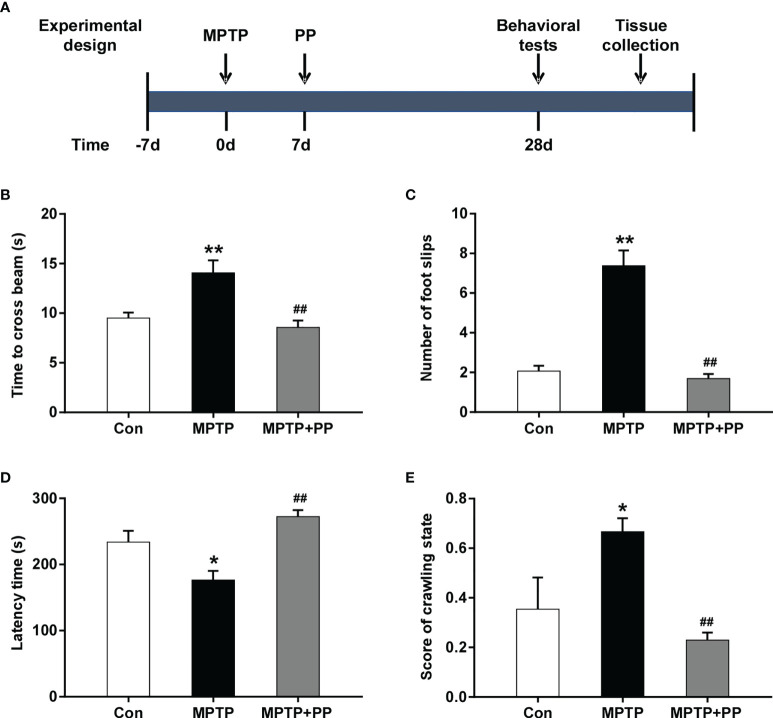
*Pediococcus pentosaceus* treatment inhibited the methyl-4-phenyl-1,2,3,6-tetrahydropyridine (MPTP)-induced motor dysfunction. **(A)** Schematic diagram of the animal experiments. **(B)** Time to cross the beam in the balance beam test. **(C)** Number of foot slips in the balance beam test. **(D)** Latency time in the rotarod test. **(E)** Score of crawling state in the pole test. Statistical comparison by one-way ANOVA with *post-hoc* comparisons of Dunnett’s multiple-comparisons test. Data are presented as means ± SEM. ^*^ *P* < 0.05 *vs*. Con group, ^**^ *P* < 0.01 *vs*. Con group, ^##^ *P* < 0.01 *vs*. MPTP group.

To evaluate the role of PP in PD, all mice were randomly divided into three groups (*n* = 8 each group), namely: Con group, MPTP group, and MPTP + PP group. The experiment was carried out after the mice were adapted to the environment for 5 days. To generate PD models, the mice were intraperitoneally injected with 25 mg/kg MPTP (MACKLIN, China) once a day for 1 week. Then, in the MPTP + PP group, the mice were intragastrically treated with 200 μl *P. pentosaceus* WMU002 (CGMCC 24884) provided by the China General Microbiological Culture Collection Center. The suspension contained 1 × 10^9^ colony-forming units/ml, which was given as 0.2 ml once a day for 4 weeks after the MPTP treatment. For the mice in the Con and MPTP groups, this was replaced with normal saline. The behavior tests in each group were performed 24 h after the last administration. TThen, the colonic contents of the mice were collected for 16s rRNA sequencing. Moreover, the brain tissues were obtained for immunohistochemistry and western blot. A diagram to describe these procedures was shown in **Figure 1A**.

### Balance beam test

The balance beam test was used to test the balance ability, muscle strength, and motor coordination of mice. The balance beam was a square with a length of 0.8 m and a width of 14 mm. Each mouse was placed on one end of the balance beam and guided to crawl to the black box for 2 days. On day 3, the mouse was placed horizontally on the beam to cross in 5 min; the number of foot slips (sliding of the front or rear paws on the smooth surface of the beam) and the time taken for the mice to cross were recorded.

### Rotarod test

Rotarod test was used to evaluate the ability of anti-fatigue and coordinated exercise of mice. The Rotarod (RL04-YLS-4D, Haifuda, Beijing, China) was equipped with automatic timers and falling sensors. The mice were trained once a day for 3 days before the experiment. On day 4, the mice were placed on an accelerated rotating cylinder, the speed of which was slowly increased from 4 to 40 revolutions per minute in 5 min. The end of the test was determined such that when the mice fell off or grabbed the device and rotated continuously for two turns without trying to walk; the latency time of the mice was measured. The test was repeated three times with an interval of at least 30 min, and the average time of latency was analyzed.

### Pole test

Pole test was used to detect the coordination ability of mice. A pole with a length of 40 cm and a diameter of 1.5 cm was fixed on a base and wrapped with non-sticky gauze for the mice to easily clamp. The mice were trained before the test to make sure that they could climb down the pole. The test ended with both hind limbs of the mice reaching the base. The total time of climbing down from the pole and the state of the mice during climbing down were measured. Each mouse was subjected to three consecutive tests, and the average was calculated for statistical analysis. The scoring criteria for the state of the mice in climbing the pole were as follows: 0, used the limbs to climb down the pole smoothly; 0.5, step-by-step spiral downward crawl, but with a sliding behavior of the hind legs; 1.0, paused several times to climb down but held on tightly to the pole; 1.5, slid down the pole and fell off; and 2.0, could not grab the pole, drop directly. After each experiment, the facility was sprayed with alcohol to get rid of the odor.

### Nissl staining

The mice were fixed in formaldehyde, embedded with paraffin, and cut into 5-μm sections for pathological staining. The slices of substantia nigra were dewaxed with xylene and rehydrated by gradient ethanol. Then, the slices were dyed with 1% tar violet dye (C0117, Beyotime, Shanghai, China) for 30 min, washed in distilled water, separated with 70% alcohol, and dehydrated by gradient alcohol. The slices were finally fixed in xylene and sealed with neutral resin. The stained tissues were observed under a microscope.

### Immunohistochemistry

The slides of substantia nigra were repaired for antigen and blocked for peroxidase. Then, the non-specific antigen of the slides was blocked with 5% fetal bovine serum for 30 min and washed with phosphate-buffered saline (PBS) three times for 5 min. The primary antibodies, including α-synuclein (1:200, BS3429, Bioworld, Minnesota, USA) and TH (1:200, AB137869, Abcam, Cambridge, UK), were incubated with the slides, respectively, at 4℃ overnight. On the second day, the slides were incubated with secondary antibody (PV-6001, ZSGB-BIO, Beijing, China) for 30 min at room temperature and washed with PBS three times for 5 min. Subsequently, the slides were added with chromogen DAB (ZLI-9018, ZSGB-BIO, Beijing, China) for target antigen detection, and the staining time was controlled under a microscope. The slides were then stained with hematoxylin dye for 2 min and dip-rinsed in hydrochloric alcohol for 2 s. After fixing by xylene and sealing with neutral resin, the brown granules in the brain tissues observed under the microscope were considered to represent a positive immune response.

### Western blot

The brain tissues of the nigrostriatum were homogenized, and the total proteins of the samples were extracted by radioimmunoprecipitation assay lysis buffer (P0013B, Beyotime, Shanghai, China) and centrifuged at 12,000 revolutions/min for 20 min, and the supernatant was taken. The protein concentration was measured with BCA kit (P0010S, Beyotime, Shanghai, China) and adjusted to the same level. After heat denaturation, the protein samples were added to 12% SDS-PAGE gel for electrophoresis, transferred to polyvinylidene fluoride membrane, and sealed with 5% skimmed milk powder solution for 2 h. Subsequently, the membrane was incubated overnight in a primary antibody of synuclein-α (1:1,000, BS3429, Bioworld, MN, USA), TH (1:1,000, BS1432, Bioworld, MN, USA), Keap1 (1:1,000, BS6783, Bioworld, MN, USA), Nrf2 (1:1,000, BS1258, Bioworld, MN, USA), SOD1 (1:1,000, BS6057, Bioworld, MN, USA), GPx1 (1:1,000, BS61511, Bioworld, MN, USA), and β-actin (1:1,000, AP0060, Bioworld, Minnesota, USA) at 4℃ and then transferred to diluent containing horseradish peroxidase-labeled secondary antibody (1:5,000, A0208, Beyotime, Shanghai, China) for further incubation. The membrane was added with chemiluminescent solution, and the gray values of each protein were recorded and analyzed by Image J software. β-actin was used as the internal reference.

### ELISA assay

The content of GABA in brain tissue was determined by ELISA (FT-T4051, Fantai, Shanghai, China). Brain tissue of the nigrostriatum and lysate were mixed at a ratio of 1:9, added with phenylmethylsulfonyl fluoride (ST506-2, Beyotime, Shanghai, China), and homogenized by a homogenizer, and then the supernatant was taken. The specific experimental operations were carried out in accordance with the instructions. The absorbance (optical density value) of each sample was measured by a microplate reader at a wavelength of 450 nm.

### Gut microbiota analysis

The colonic contents of mice were collected and frozen at -80℃, and DNA was extracted with DNA extraction kit (QIAamp DNA stool mini kit, Qiagen, Hilden, Germany) for real-time fluorescence quantitative polymerase chain reaction (PCR), which amplified the V3–V4 region of the bacterial 16s rRNA gene. The purified amplicons were sequenced by Illumina MiSeq system, and the high-quality sequences were clustered according to 97% similarity by *de novo* UCLUST algorithm to obtain operational taxonomic unit (OTU). Then, the α-diversity index was assessed, and the difference of OTUs was analyzed by Mann–Whitney nonparametric test. Linear discriminant analysis (LDA) effect size (LEfSe) analysis was used to identify differential marker species by LDA algorithm.

### Statistical analysis

All data were expressed as the mean ± standard error of mean (SEM) and were analyzed by using GraphPad Prism 7 software (La Jolla, CA, USA), in which one-way ANOVA was used for the overall variance analysis, and Dunnett’s multiple-comparisons test was used to compare the differences between groups. The composition difference of gut microbiota was analyzed by Kruskal–Wallis rank sum test, and *post-hoc* test was performed to compare the differences among the three groups by Tukey–Kramer. *P <*0.05 was considered statistically significant.

## Results

### PP treatment inhibited the MPTP-induced motor dysfunction

In the balance beam test, the time to cross the beam and the number of foot slips in the MPTP group were significantly increased compared with the Con group (*P* < 0.01, **Figures 1B, C**), while these were reversed after PP treatment (*P* < 0.01, **Figures 1B, C**). In the rotarod test, the latency time of the MPTP group was significantly shorter than that of the Con group (*P* < 0.05, **Figure 1D**), while the latency time of the MPTP + PP group was longer than that of the MPTP group (*P* < 0.01, **Figure 1D**). In the pole test, the score of the crawling state in the MPTP group was significantly increased compared with the Con group (*P* < 0.05, **Figure 1E**), whereas the score in the MPTP + PP group was significantly decreased compared with the MPTP group (*P* < 0.01, **Figure 1E**). These results suggested that PP could improve the MPTP-induced motor dysfunction.

### PP treatment suppressed the MPTP-induced neuronal degeneration

**Figure 2:**
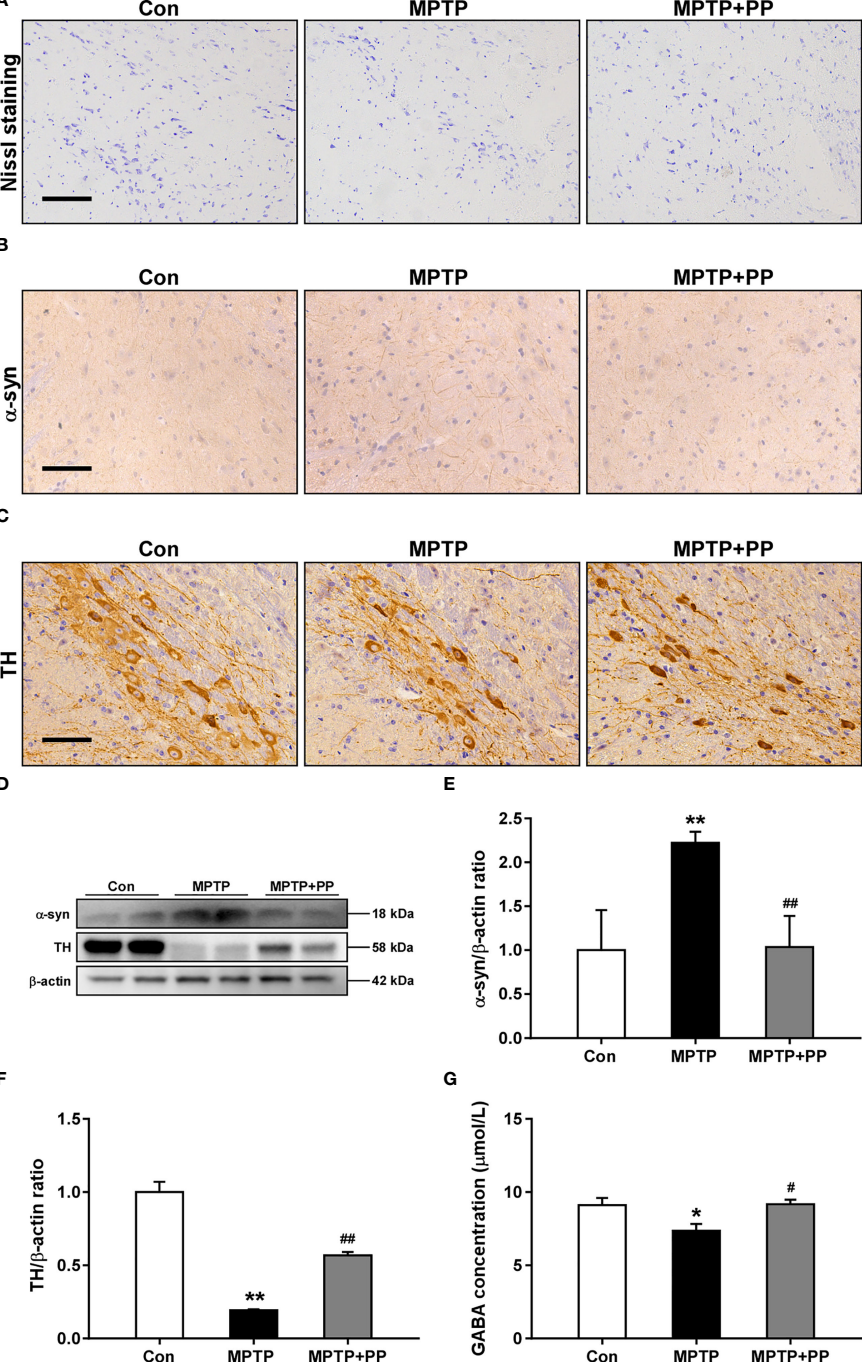
*Pediococcus pentosaceus* treatment attenuated the methyl-4-phenyl-1,2,3,6-tetrahydropyridine (MPTP)-induced neuronal degeneration and the decreased level of γ-aminobutyric acid (GABA). **(A)** Representative images of Nissl staining for Nissl body in neurons. Magnification, ×400; scale bar = 100 μm. **(B)** Representative immunohistochemistry images of α-synuclein in substantia nigra. Magnification, ×400; scale bar = 100 μm. **(C)** Representative immunohistochemistry images of TH in substantia nigra. Magnification, ×400; scale bar = 100 μm. **(D)** Representative Western blotting images of α-synuclein (α-syn) and TH. **(E)** Quantitative analysis of α-syn. **(F)** Quantitative analysis of TH. β-actin was used as the internal reference. **(G)** Quantitative analysis of the GABA level in the brain. Statistical comparison by one-way ANOVA with *post-hoc* comparisons of Dunnett’s multiple-comparisons test. Data are presented as means ± SEM; *n* = 4–6 per group. ^*^ *P* < 0.05 *vs*. Con group, ^**^ *P* < 0.01 *vs*. Con group, ^#^ *P* < 0.05 *vs*. MPTP group, ^##^ *P* < 0.01 *vs*. MPTP group.

The results of Nissl staining showed that the number of neurons in the MPTP group was less than that in the Con group, while the number of neurons in the MPTP + PP group was more than that in the MPTP group (**Figure 2A**), suggesting that PP treatment reduced the neuronal damage of PD. Then, we assessed the level of α-synuclein in substantia nigra by immunohistochemistry and Western blot. The results showed that the accumulation of α-synuclein in the MPTP group was significantly increased compared with that in the Con group, while the α-synuclein level was decreased after PP treatment (*P* < 0.01, **Figures 2B–E**). Moreover, the TH level was measured to evaluate the injury of dopaminergic neurons. The results showed that the TH level of the substantia nigra in the MPTP group was significantly lower than that in the Con group, while the TH level in the MPTP + PP group was significantly higher than that in the MPTP group (*P* < 0.01, **Figures 2C, D, F**). These results suggested that PP treatment could attenuate the dopaminergic neuronal degeneration of PD.

### PP treatment reversed the MPTP-induced decreased level of GABA

Subsequently, we measured the concentration of GABA in the mouse brain. The result showed that the GABA level was decreased in MPTP mice than that in the Con group, while the GABA level in the brain was increased after PP treatment (*P* < 0.05, **Figure 2G**), suggesting that PP could reverse the decrease of GABA in PD.

### PP treatment improved the MPTP-induced oxidative stress and regulated the levels of Keap1 and Nrf2

**Figure 3:**
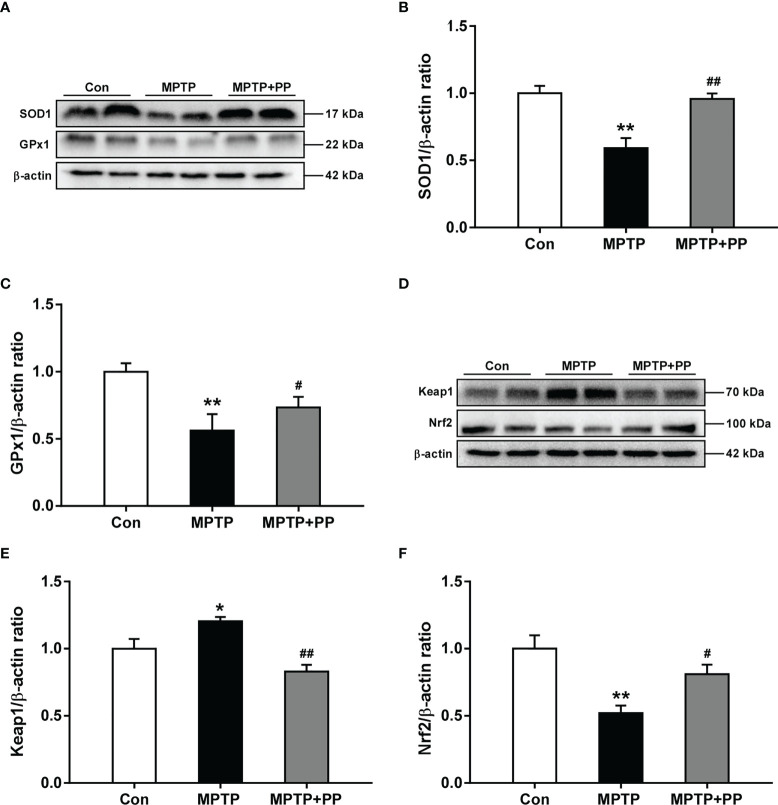
*Pediococcus pentosaceus* treatment improved the methyl-4-phenyl-1,2,3,6-tetrahydropyridine (MPTP)-induced oxidative stress and regulated the levels of Keap1 and Nrf2. **(A)** Representative western blotting images of SOD1 and GPx1. **(B)** Quantitative analysis of SOD1. **(C)** Quantitative analysis of GPx1. **(D)** Representative western blotting images of Keap1 and Nrf2. **(E)** Quantitative analysis of Keap1. **(F)** Quantitative analysis of Nrf2. β-actin was used as internal reference. Statistical comparison by one-way ANOVA with *post-hoc* comparisons of Dunnett’s multiple-comparisons test. Data are presented as means ± SEM; *n* = 4–6 per group. ^*^ *P* < 0.05 *vs*. Con group, ^**^ *P* < 0.01 *vs*. Con group, ^#^ *P* < 0.05 *vs*. MPTP group, ^##^ *P* < 0.01 *vs*. MPTP group.

The activities of the antioxidant enzyme SOD1 decreased significantly in the MPTP group compared with those in the Con group, while the SOD1 level in the MPTP + PP group was significantly increased than that in the MPTP group (*P* < 0.01, **Figures 3A, B**). Similarly, the activities of the antioxidant enzyme GPx1 decreased significantly in the MPTP group compared with the Con group (*P* < 0.01, **Figures 3A, C**), which was reversed after PP treatment (*P* < 0.05, **Figures 3A, C**). Furthermore, we measured the expression level of Nrf2 signal pathway-related proteins, including Keap1 and Nrf2. The results showed that the expression level of Keap1 was significantly increased in the MPTP group compared with the Con group (*P* < 0.05, **Figures 3D, E**), while the level was significantly decreased in the MPTP + PP group compared with the MPTP group (*P* < 0.01, **Figures 3D, E**). On the contrary, the Nrf2 level was decreased significantly in the MPTP group (*P* < 0.01, **Figures 3D, F**), whereas the level was elevated after PP treatment (*P* < 0.05, **Figures 3D, F**). These results suggested that PP treatment could improve oxidative stress by regulating the abnormal Nrf2 signaling pathway in PD.

### PP treatment reversed the MPTP-induced abnormal microbiota composition

**Figure 4:**
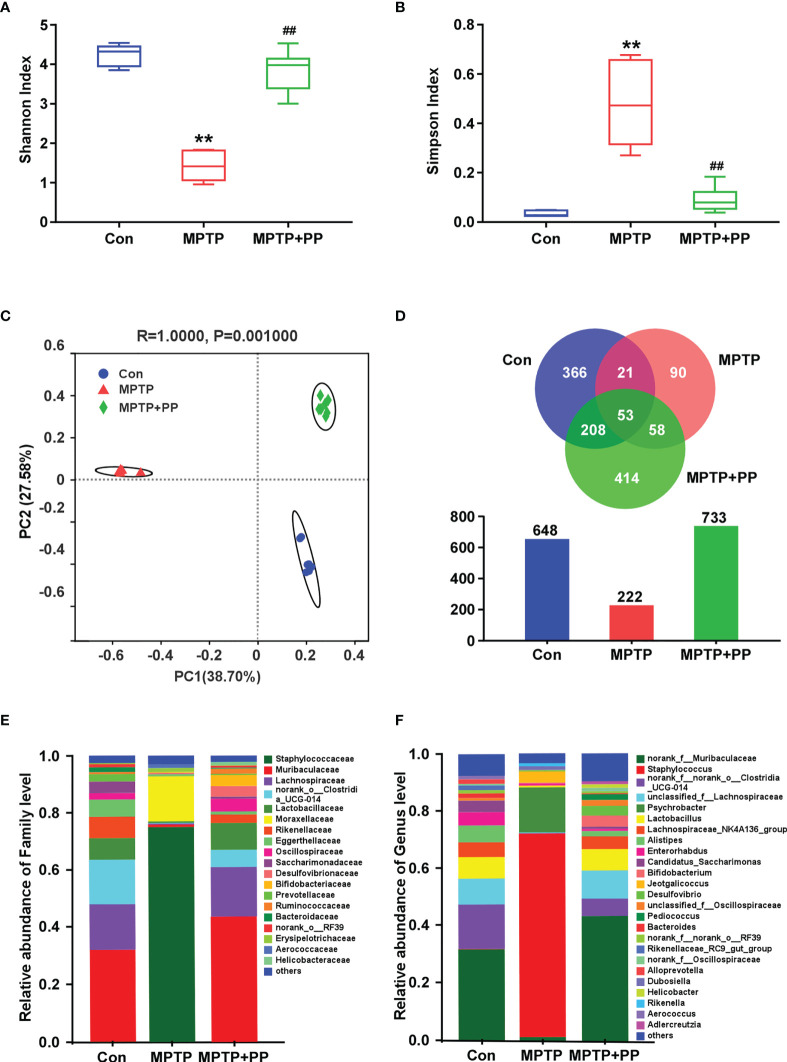
*Pediococcus pentosaceus* treatment reversed the methyl-4-phenyl-1,2,3,6-tetrahydropyridine (MPTP)-induced abnormal microbiota composition. **(A)** Shannon index of gut microbial diversity. **(B)** Simpson index of gut microbial diversity. **(C)** Principal coordinate analysis plots of gut microbial diversity. **(D)** Venn diagram and quantitative analysis of microbial community composition among three groups. **(E)** Relative abundance of gut microbiota at the family level among three groups. **(F)** Relative abundance of gut microbiota at the genus level among three groups. Statistical comparison by Kruskal–Wallis rank sum test with *post-hoc* comparisons of Tukey–Kramer. Data are presented as median + interquartile range. ^**^ *P* < 0.01 *vs*. Con group, ^##^ *P* < 0.01 *vs*. MPTP group.

**Figure 5:**
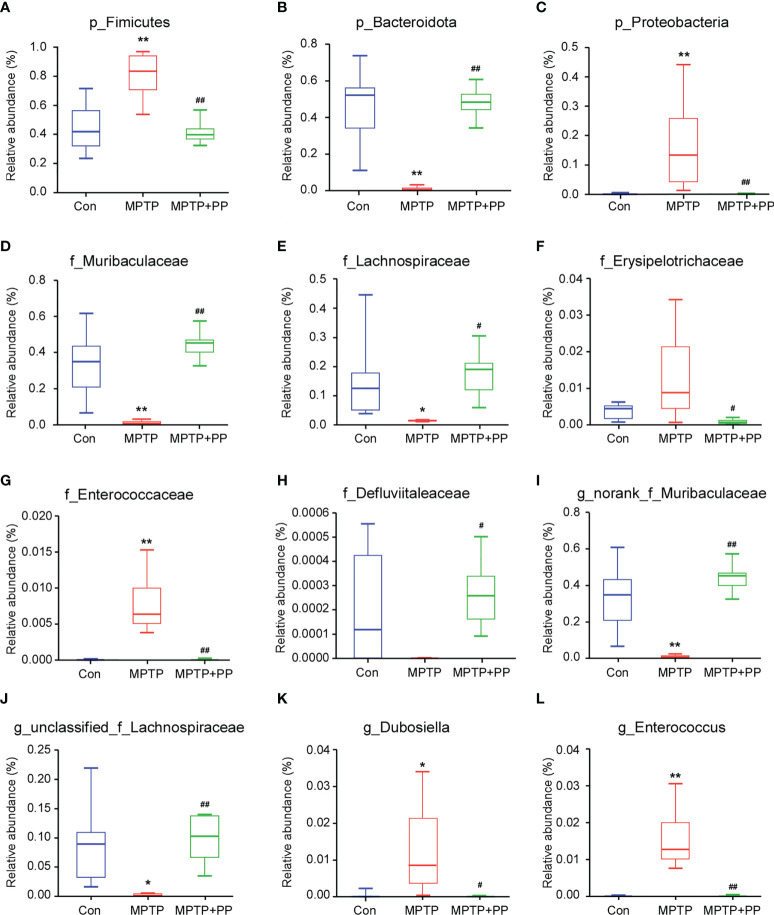
*Pediococcus pentosaceus* treatment reversed the methyl-4-phenyl-1,2,3,6-tetrahydropyridine (MPTP)-induced change of the relative abundance of different bacteria. **(A)** p_*Firmicutes*, **(B)** p_*Bacteroidota*, **(C)** p_*Proteobacteria*, **(D)** f_*Muribaculaceae*, **(E)** f_*Lachnospiraceae*, **(F)** _*Erysipelotrichaceae*, **(G)** f_*Enterococcaceae*, **(H)** f_*Defluviitaleaceae*, **(I)** g_norank_f_*Muribaculaceae*, **(J)** g_unclassified_f_*Lachnospiraceae*, **(K)** g_*Dubosiella*, and (**L**) g_*Enterococcus*. Statistical comparison by Kruskal–Wallis rank sum test with *post-hoc* comparisons of Tukey–Kramer. Data are presented as median + interquartile range. ^*^ *P* < 0.01 *vs*. Con group, ^**^ *P* < 0.01 *vs*. Con group, ^#^ *P* < 0.01 *vs*. MPTP group, ^##^ *P* < 0.01 *vs*. MPTP group.

**Figure 6:**
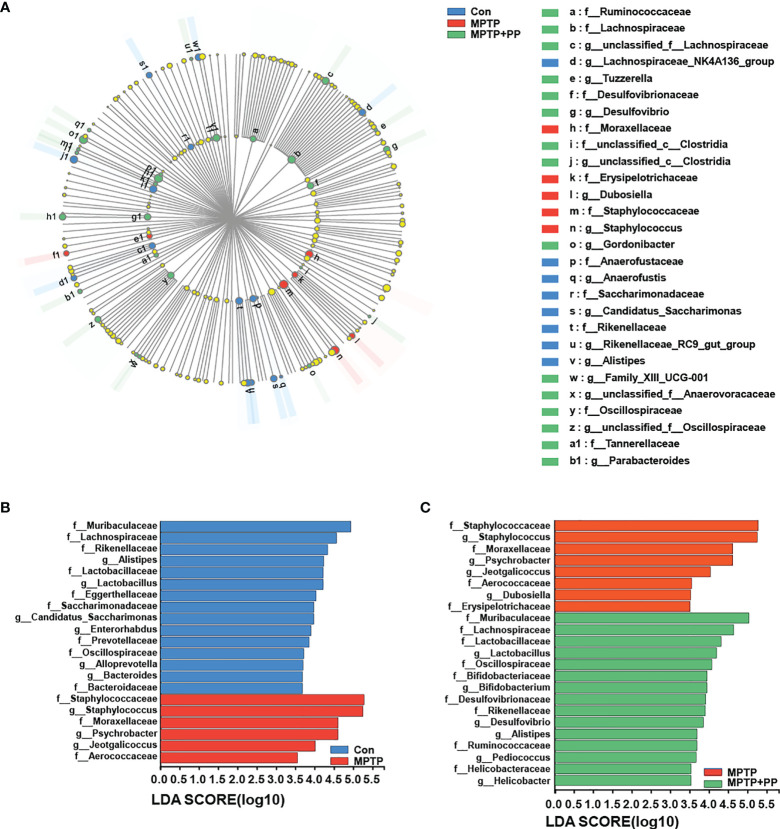
*Pediococcus pentosaceus* (PP) treatment changed the methyl-4-phenyl-1,2,3,6-tetrahydropyridine MPTP)-induced abnormal composition of specific bacterial taxa. **(A)** LEfSe multilevel species hierarchy tree cladogram representing the microbial structure and the predominant bacteria among the three groups. **(B)** Microbial structure and the predominant bacteria between the Con group and MPTP group; linear discriminant analysis (LDA) > 3.5. **(C)** Microbial structure and the predominant bacteria between the MPTP group and the MPTP + PP group; LDA > 3.5.

We used the Shannon index and the Simpson index to assess the changes in gut microbiota α-diversity of mice. The results showed that the Shannon index in the MPTP group was significantly decreased compared with the Con group, while the index in the MPTP + PP group was significantly increased compared with the MPTP group (*P* < 0.01, **Figure 4A**). On the contrary, the Simpson index in the MPTP group was significantly higher than the Con group, while the index in the MPTP + PP group was significantly decreased than the MPTP group (*P* < 0.01, **Figure 4B**), suggesting that PP could significantly improve the decline of microbial diversity of PD. Meanwhile, the principal coordinate analysis on amplicon sequence variant (ASV) level showed the changes of microbial β-diversity, which was significantly different among the three groups (**Figure 4C**). The Venn diagram of microbiota showed that 53 ASVs were shared among the three groups, whereas 208 ASVs were shared only between the Con group and the MPTP + PP group (**Figure 4D**). The microbial community composition was primarily composed of *Staphylococcaceae*, *Muribacullaceae*, and *Lachnospiraceae* (**Figures 4E, F**). Then, we measured the different bacteria in each group. At the phylum level, the relative abundance of *Firmicutes* and *Proteobacteria* in the MPTP group was increased than the Con group, whereas the relative abundance of which was decreased in the MPTP + PP group compared with the MPTP group (*P* < 0.01, **Figures 5A, C**). In contrast, phylum *Bacteroidota* in the MPTP group was decreased compared with the Con group and was increased in the MPTP + PP group (*P* < 0.01, **Figure 5B**). At the family level, the relative abundance of *Muribaculaceae* (*P* < 0.01, **Figure 5D**), *Lachnospiraceae* (*P* < 0.05, **Figure 5E**), and *Defluviitaleaceae* (*P* > 0.05, **Figure 5H**) in the MPTP group was decreased than the Con group, whereas the relative abundance of *Muribaculaceae* (*P* < 0.01, **Figure 5D**), *Lachnospiraceae* (*P* < 0.05, **Figure 5E**), and *Defluviitaleaceae* (*P* < 0.05, **Figure 5H**), respectively, was increased in the MPTP + PP group compared with the MPTP group. Additionally, the relative abundance of *Erysipelotrichaceae* (*P* > 0.05, **Figure 5F**) and *Enterococcaceae* (*P* < 0.01, **Figure 5G**), respectively, at the family level in the MPTP group was increased compared with the Con group, whereas the relative abundance of *Erysipelotrichaceae* (*P* < 0.05, **Figure 5F**) and *Enterococcaceae* (*P* < 0.01, **Figure 5G**) at the family level was decreased after PP treatment. At the genus level, the relative abundance of norank_f_*Muribaculaceae* (*P* < 0.01, **Figure 5I**) and *Lachnospiraceae* (*P* < 0.05, **Figure 5J**) in the MPTP group was decreased than that of the Con group, whereas the relative abundance of norank_f_*Muribaculaceae* (*P* < 0.01, **Figure 5I**) and *Lachnospiraceae* (*P* < 0.01, **Figure 5J**) was increased in the MPTP + PP group compared with the MPTP group. Moreover, the relative abundance of *Dubosiella* (*P* < 0.05, **Figure 5K**) and *Enterococcus* (*P* < 0.01, **Figure 5L**) at the genus level in the MPTP group was increased compared with the Con group, whereas the relative abundance of *Dubosiella* (*P* < 0.05, **Figure 5K**) and *Enterococcus* (*P* < 0.01, **Figure 5L**) was decreased after PP treatment. Subsequently, we further identified the specific bacterial taxa among the three groups by LEfSe analysis. The cladogram represented the microbial structure and predominant bacteria among the three groups from the family level to the genus level (**Figure 6**). These results suggested that the microbial composition in PD was significantly changed, while PP could improve the abnormal microbial composition of PD.

## Discussion

Oxidative stress was involved in the pathogenesis of PD. Recently, the anti-PD effect of probiotics was related to the antioxidant effect of its metabolites and the regulation of the gut–brain axis. In this study, we demonstrated that PP (a GABA-producing bacteria) treatment significantly improved the MPTP-induced motor dysfunction, neuronal degeneration, and oxidative stress in mice. Moreover, PP treatment could reverse the abnormal gut microbiota, increase the level of GABA, and regulate the levels of the Nrf2 pathway-related proteins in the brain of MPTP-induced mice. The neuroprotective mechanism of PP on PD might be related to the regulation of the metabolite GABA–gut–brain axis.

Motor dysfunction was characteristic of PD (Feng et al., 2020). PD patients showed typical symptoms of motor dysfunction (Sarasso et al., 2021), which was caused by the loss of dopaminergic neurons in the substantia nigra pars compacta (Surmeier, 2018) and widespread α-synuclein aggregated in the form of Lewy bodies (Raza et al., 2019). Mounting studies showed that the loss of dopaminergic neurons and the α-synuclein level increased in PD animal models (Su et al., 2019; Zhao et al., 2021). In this study, we proved that PP treatment could improve the MPTP-induced motor disorders and decrease the MPTP-induced dopaminergic neuronal loss and α-synuclein accumulation, suggesting that PP could improve the neurodegeneration of PD.

Oxidative stress played an important role in the progression of PD, aggravated the destruction of oxidoreductive homeostasis, and induced the progressive neurodegeneration of dopaminergic neurons. It was reported that PD patients exhibited obvious oxidative stress, manifested as the increased concentrations of DNA oxidative damage and lipid peroxidation markers in the blood as well as the decreased levels of antioxidant substances, such as catalase and glutathione (Wei et al., 2018). A large number of studies had shown that inhibiting the excessive oxidative stress could improve PD (Lv et al., 2020; Yan et al., 2021). In this study, PP treatment significantly increased the levels of antioxidant enzymes SOD and GPx in PD. Similarly, *P. pentosaceus* ZJUAF-4 increased the activity of SOD, while it reduced the production of ROS and MDA and relieved oxidative stress injury in mice induced by quinoline (Hao et al., 2021). Additionally, *Lactobacillus pentosus*, one of the GABA-producing bacteria, significantly decreased the intracellular ROS and inhibited particulate matter-induced cell death (Lee and Park, 2021). *Lactobacillus paracasei* PS23 could also upregulate the expression level of antioxidant genes, improve the activity of SOD, and alleviate the motor and anxiety behavior in aging mice (Cheng et al., 2022). Our results indicated that PP was able to alleviate the oxidative stress of PD.

Nrf2, a redox-regulated transcription factor, was critically involved in the regulation of oxidative stress in PD (He et al., 2020; Parga et al., 2021). Normally, Nrf2 bound to Keap1 in the cytoplasm, while in excess of ROS, Nrf2 disconnected from Keap1, migrated to the nucleus, and combined with ARE, which resulted in the upregulation of cytoprotective and antioxidant enzymes that protected against oxidative stress (Fão et al., 2019). It was reported that Nrf2 transcription and protein expression were increased in PD patients compared with the control, and the level of Nrf2 transcription was directly related to the course of the disease (Petrillo et al., 2020). Accumulating evidence showed that the activation of the Nrf2 signaling pathway was significantly inhibited in MPTP-induced mice (Lee et al., 2018; Mohamed et al., 2021), while inducing Nrf2 activation could mitigate the degeneration of dopaminergic neurons and provide neuroprotection for PD (Zhang et al., 2021). Our results showed that PP treatment could increase the level of Nrf2, thus reducing the expression level of Keap1, which was consistent with former studies. PP treatment increased the Nrf2 level and its downstream genes as well as restored redox homeostasis in quinoline-induced mice (Hao et al., 2021). In addition, *Lactobacillus plantarum* KSFY06 downregulated the Keap1 expression and upregulated the Nrf2 mRNA expression in D-galactose-induced mice (Li et al., 2021). *Lactobacillus plantarum* DP189 could upregulate the Nrf2 level and the mRNA level of antioxidant enzymes (Wu et al., 2021), which further improved the neurodegeneration in PD (Wang et al., 2022). Our results suggested that PP could regulate the Nrf2 pathway in exerting an antioxidant role in PD.

The composition and the structure of the gut microbiota in PD patients and animals were disturbed (Sun et al., 2018; Lin et al., 2019). In this study, gut microbiota diversity was decreased, and the relative abundance of *Firmicutes* and *Proteobacteria* was increased in PD, which was reversed by PP treatment. Consistent with our results, a higher abundance of *Firmicutes* and phylum *Proteobacteria* in the fecal samples of PD mice was observed (Sun et al., 2018; Zhou et al., 2019). In this study, the decline of *Bacteroidota* was restored after PP treatment. Previous studies confirmed that *Bacteroidota* was reduced in PD patients compared with the matched controls (Unger et al., 2016). The abundance of *Bacteroidota* was significantly positively correlated with gut barrier function and cognitive behavior index but negatively correlated with hippocampal inflammation (Shi et al., 2020). In this study, PP reversed the low abundance of *Muribaculaceae*, *Lachnospiraceae*, and *Defluviitaleaceae* as well as the overabundance of *Erysipelotrichaceae*, *Enterococcaceae*, *Dubosiella*, and *Enterococcus* of PD. It was demonstrated that *Lachnospiraceae* and the related genera were decreased in PD patients (Barichella et al., 2019; Vascellari et al., 2020), while *Enterococcaceae* was increased in patients and mice of PD (Lai et al., 2018; Pietrucci et al., 2019), and a lower level of *Lachnospiraceae* and a higher level of *Enterobacteriaceae* were also correlated with the aggravation of motor impairment and disease severity (Barichella et al., 2019; Pietrucci et al., 2019). Our results suggested that PP could regulate the homeostasis of gut microbiota.

GABA was an inhibitory neurotransmitter which could effectively reduce the excitability of neurons and maintain the redox homeostasis of cells (Sarasa et al., 2020). A study indicated that an abnormal GABA level was positively correlated with the severity of motor symptoms in PD (O'Gorman Tuura et al., 2018). A clinical study showed that the level of GABA in the brain of PD patients was significantly lower than that of healthy controls (Song et al., 2021), which resulted in the dopaminergic pathology of PD. GABA treatment increased the expression level of Nrf2 and the activity of antioxidant enzymes, such as CAT and SOD, effectively reducing the consumption of glutathione and the level of ROS and thereby reducing oxidative stress (Zhu et al., 2019; Choe et al., 2021). Excessive GABA in astrocytes caused the loss of TH, which showed a decrease in the discharge of dopaminergic neurons, while blocking the astrocytic GABA synthesis could reverse this effect (Heo et al., 2020). GABA is generally considered as a key candidate mediator, which is a metabolite of the functional bacteria. GABA might be involved in the communication of the gut–brain axis and influence brain function. We believed that PP might possess many mechanisms in the neuroprotection of PD, among which GABA might be a very important mechanism. The level of GABA affected by symbiotic gut microbiota could lead to behavioral and cognitive changes (Strandwitz, 2018; Zheng et al., 2019), which were consistent with our results. Furthermore, oral supplementation of *Lactobacillus plantarum* strain SNK12 upregulated the GABA level and improved the habitual ability and stress behavior in stress-induced mice (Tsukahara et al., 2019). Long-term treatment with *Lactobacillus rhamnosus* JB-1 influenced the mRNA expressions of GABA receptors in the brain, regulated the gut–brain axis in a vagal-dependent manner, and reduced the depression- and anxiety-like behaviors of pressure-induced mice (Bravo et al., 2011). The treatment of probiotics and prebiotics also effectively restored the level of GABA and normalized the levels of lipid peroxidation and antioxidant glutathione in the brain of rats with cerebral poisoning (Al Suhaibani et al., 2021), indicating that GABA produced by the gut microbiota had great potential in regulating oxidative stress associated with neurodegenerative diseases. Given that gut microbiota has been shown to be influenced by PP and that the level of cerebral GABA was altered significantly, the GABA/Nrf2 pathway might be changed by the communication between PP and the gut–brain axis. The neuroprotective mechanism of PP on the oxidative stress of PD through the metabolites of the GABA–gut–brain axis needs to be studied further.

Collectively, our results illuminated that PP treatment had an antioxidant effect in MPTP-induced mice, and its beneficial mechanism was referred to in the GABA/Nrf2 pathway *via* adjusting the gut–brain axis. PP was able to be a promising candidate used for PD treatment due to the metabolite of GABA.

## Data availability statement

The datasets presented in this study can be found in online repositories. The names of the repository and accession number can be found below: NCBI, SUB11968017. The SRA records can be accessible with the following link: https://www.ncbi.nlm.nih.gov/sra/PRJNA873227.

## Ethics statement

The animal study was reviewed and approved by the First Affiliated Hospital of Wenzhou Medical University.

## Author contributions

JL and YaZ conceived and designed the experiments. SP, HW, SY, YK, HY, YuZ, XC, and WC performed the experiments and conducted the statistical analyses. All authors contributed to the article and approved the submitted version.

## Funding

This work was supported by the Natural Science Foundation of Zhejiang Province (LGD22H090011) and Wenzhou Science and Technology Research Funds (Y20220816).

## Conflict of interest

The authors declare that the research was conducted in the absence of any commercial or financial relationships that could be construed as a potential conflict of interest.

## Publisher’s note

All claims expressed in this article are solely those of the authors and do not necessarily represent those of their affiliated organizations, or those of the publisher, the editors and the reviewers. Any product that may be evaluated in this article, or claim that may be made by its manufacturer, is not guaranteed or endorsed by the publisher.
